# Rare Combinational Hemoglobinopathies

**DOI:** 10.7759/cureus.32327

**Published:** 2022-12-08

**Authors:** Ammar Husan, Sam N Amarasinghe, Andee Fontenot, Muhammad W Khan

**Affiliations:** 1 Family Medicine, Louisiana State University Health Sciences Shreveport, Shreveport, USA; 2 Medical School, Louisiana State University Health Sciences Shreveport, Shreveport, USA; 3 Neurology, University of Rochester, Rochester, USA

**Keywords:** d-punjab, korle-bu, sickle cell, anemia, hemoglobinopathy

## Abstract

Hemoglobinopathies are genetic defects that result in the abnormal formation and composition of globin chains in the hemoglobin molecule. Sickle cell disease is one of the more common forms of genetic malformation, while Hemoglobin (Hb) Arab, Lepore, Korle-Bu, Kansas, D-Punjab, and Hasharon are uncommon presentations. Herein, we describe the case of a young patient who presented with a low hemoglobin level and was subsequently diagnosed with a rare combination of Hemoglobin Korle-Bu, D-Punjab, and sickle cell trait.

## Introduction

Hemoglobin (Hb) is a globular protein that carries oxygen from the lungs to body tissue for its metabolic demands. Hb is located in erythrocytes and is composed of heme, a central iron molecule, and four globin subunits. The combination of four of the six types of globin subunits (beta (β), delta (δ), gamma (γ), epsilon (ε), alpha (α), and zeta (ζ)) determines the hemoglobin type. Physiologically normal Hb molecules include hemoglobin A1 (HbA1) which comprises two α and two β subunits, hemoglobin A2 (HbA2) which comprises two α and two δ subunits, and fetal hemoglobin (HbF) which comprises two α and two γ subunits [1].

Depending on an individual's genetic code, different Hb molecule dysfunctions can arise. The dysfunctioning molecule may be detrimental, causing a variety of symptomatic presentations or it can be clinically silent. Some of these dysfunctions are a result of reduced globin expression as in the case of thalassemia. Thalassemia is caused by reduced expression of either the α or β globin chain of Hb. α-thalassemia is most often caused by a deletion, while β-thalassemia is most often caused by a point mutation [2]. There are also situations where globin expression is not affected, but rather a structural mutation of the Hb molecule results in the pathologic state. These hemoglobinopathies result from mutated globin chains that cause structurally altered Hb molecules. While much is known about the most common hemoglobin variant in the world HbS, there are other rare variants including Hb D-Punjab and Hb Korle-Bu [3]. Genetic variations of Hb are an inherited single-gene disorder frequently found in ethnic populations of Africa, Southeast Asia, and the Mediterranean, most commonly passed down as autosomal dominant traits [4]. Though several variants may not cause any pathology or anemia, and often may not be classed as hemoglobinopathies, these mutations are broadly classified as pathological or non-pathological entities depending on the symptoms they may produce in the affected population. When hemoglobinopathies are suspected in a patient, electrophoresis, isoelectric focusing, high-performance liquid chromatography (HPLC), and molecular testing are used to screen and diagnose [2].

## Case presentation

A female of African American descent in her early twenties presented to the emergency department with complaints of abdominal pain that started several hours prior to her arrival. She stated that the pain was located in her lower abdomen, characterized it as dull, and specified that it did not radiate. The abdominal pain was said to have had an insidious onset and progressively worsened. The patient denied noticing any aggravating or relieving factors. Although she was not menstruating at the time of presentation, she had a past medical history of abnormal uterine bleeding. She had not experienced any trauma to the affected area prior to the onset of symptoms, nor did she have a history of associated constipation, diarrhea, vomiting, urine retention, hematochezia, fever, numbness, tingling, or weakness of the extremities. She denied chest pain with exertion, productive cough, and shortness of breath. She had no prescribed medications and no known drug allergies. Her family history was unknown as the patient was adopted. 

Physical examination at the time of arrival was significant for tenderness at lower abdominal quadrants bilaterally. Vital signs were only significant for tachycardia. Right upper quadrant ultrasound was negative for gallbladder pathology. Computed tomography (CT) of the chest, abdomen, and pelvis showed no evidence of appendicitis or other abnormalities. Laboratory tests collected included a complete blood count (CBC), a comprehensive metabolic panel (CMP), lipase level, troponin levels, pregnancy test, and blood cultures. Obstetrics/gynecology was consulted due to her past medical history of abnormal uterine bleeding. A full gynecological physical exam and pap smear showed no abnormalities in addition to a negative pregnancy test. CBC was significant for Hb of 8.2 g/dL, hematocrit (Hct) of 31%, red cell distribution width (RDW) of 16%, mean corpuscular volume of 73 fL, and white blood cell count (WBC) of 6x10^9^/L. CMP, lipase, and troponin were within normal limits. Blood cultures resulted in no growth. 

**Figure 1:**
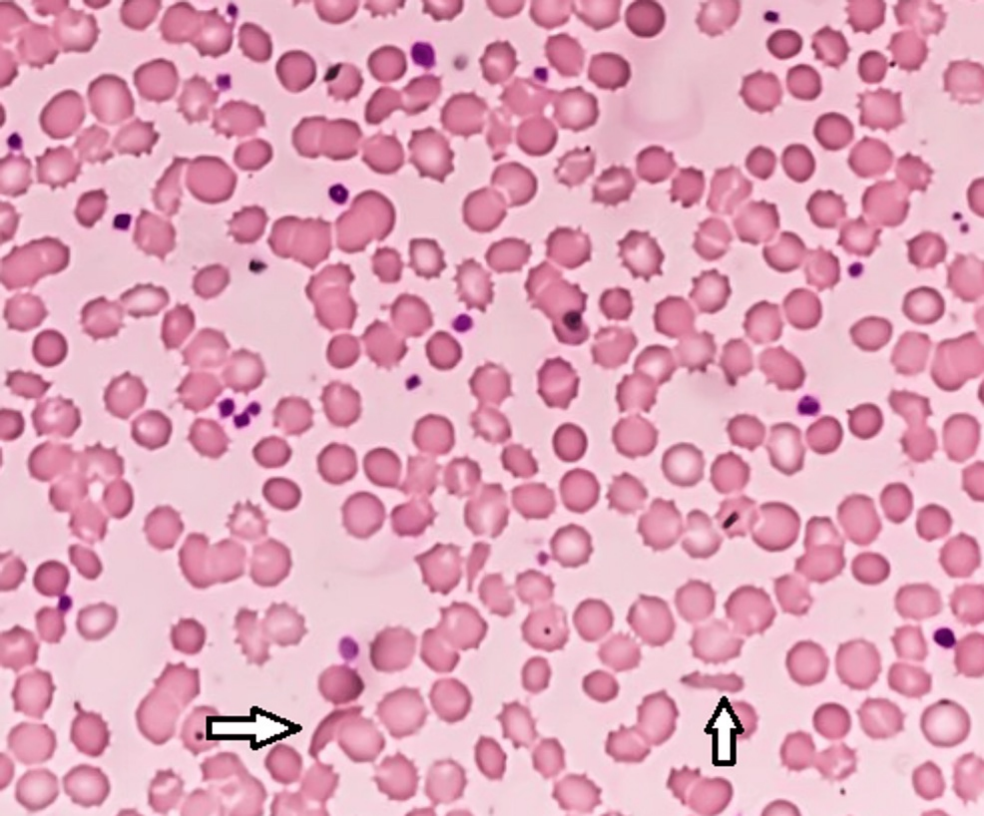
Peripheral blood smear with hematoxylin and eosin staining. The left arrow is pointing at a sickle cell and the right arrow is pointing at an echinocyte. Drepanocytes are also present but not labeled with an arrow.

Repeat blood work was obtained for histopathology with hematoxylin and eosin (H&E) staining to further evaluate the low Hb level that did not seem to correlate with any particular illness or be associated with objective pathologic evidence on radiological imaging and detailed physical examinations. Normal red blood cell (RBC) morphology is in a biconcave shape which increases the surface area to volume ratio to aid in gas exchange and improve mobility through blood vessels. This patient’s histopathology demonstrated poikilocytosis, or abnormal RBC morphology. Specifically, her blood smear showed echinocytes, dacrocytes, and sickle cells amongst normal RBCs (Figure 1). Echinocytes have a smooth rounded shape with cytoplasmic projections while dacrocytes are teardrop-shaped. Lastly, sickle cells, or drepanocytes, are crescent-shaped [1]. These abnormal morphologies are often associated with hypoxia, oxidative stress, or abnormal hemoglobin production and variants. Clinically, these abnormal RBCs can cause Hb levels to drop, decrease oxygenation of tissues, and potentially cause clotting due to difficulty moving through small vessels. Based on this patient’s blood smear, thalassemia or sickle cell trait/disease was initially suspected due to the dacrocytes and sickle cells, respectively. Hb electrophoresis was then performed and showed no evidence of HbA_1_, HbA_2_, or HbF. However, electrophoresis did show evidence of HbS at 53%, Hb Korle-Bu at 17%, and Hb D-Punjab at 30%. 

The patient’s abdominal pain soon subsided in the absence of any active medical or surgical intervention besides supportive care, and she was discharged from the hospital. The cause of this patient's initial abdominal pain was hypothesized to be due to a minor sickle cell pain crisis with her blood smear results in mind. The quick resolution of the abdominal pain was most likely due to rest and adequate rehydration. Outpatient follow-up with a primary care physician and referral to hematology/oncology was arranged. Unfortunately, the patient did not show up for her scheduled outpatient hematologist appointment and was ultimately lost to follow-up.

## Discussion

Newborn screening for early detection of hemoglobinopathies has helped decrease morbidity and mortality in non-endemic countries like the United States. In many endemic countries, newborn screening is not routinely performed. Instead, these countries rely on premarital and prenatal screening for earlier detection and prevention [5]. With the rise of immigration between endemic and non-endemic countries over the last several decades, the prevalence of hemoglobinopathies has increased in otherwise non-endemic countries. In addition, immigrants from an endemic country are more likely to marry individuals from the same country or culture, leading to a higher incidence of affected children when parents are asymptomatic carriers [6]. While expectant parents with proper access to healthcare in the United States may be able to obtain carrier and prenatal screening, medically underserved populations such as certain immigrant groups are less likely to undergo screening or receive any preventative treatment [5-7].

**Table 1:** Hemoglobinopathy variant comparison

	Heterozygous	Homozygous or multiple hemoglobinopathies
Age of Presentation	Typically asymptomatic	3-6 months
Clinical Features	If symptomatic: Painless hematuria, hyposthenuria, vaso-occlusive events with severe oxygen deficiency	Vaso-occlusive crisis, dactylitis, stroke, acute chest syndrome, acute hemolytic crisis
RBC Morphology	If symptomatic: drepanocytes, target cells, Howell-Jolly bodies	Drepanocytes, target cells, Howell-Jolly bodies

HbS is a variant that results from a point mutation in the β globin gene (GTG→GAG) at position 6. Consequently, valine is produced instead of glutamic acid. Sickle cell trait is diagnosed when a patient has one normal β globin gene and one copy of the mutated β globin gene while sickle cell disease is associated with two copies of the mutated β globin gene. Clinically, sickle cell trait is usually asymptomatic. Painless gross hematuria from renal papillary necrosis is often the only symptom. Extreme physical exertion can cause a clinically similar picture to sickle cell disease. Sickle cell disease can have severe clinical complications including vaso-occlusive events such as dactylitis in children, vaso-occlusive crisis, acute chest syndrome, stroke, and acute hemolytic crisis [8]. 

Several studies have been conducted in relation to the incidence of HbS and its increasing prevalence in populations. HbS inherited in the heterozygous form, or sickle cell trait, was found to originate and prevail as a form of evolutionary benefit predominantly in malaria-endemic countries [9].

HbD is a variant that results from a mutated β globin gene. Hemoglobin D-Punjab, also known as D-Los Angeles, is a specific variant of Hb D that was first discovered in a Los Angeles-based family with Indian, British, and American features in 1951 [10]. In 1962, multiple molecular structure variants of HbD were discovered and analyzed. Each variant was named for the region in which it was observed including Hb D-Chicago, Hb D-North Carolina, Hb D-Punjab, Hb D-Portugal, and Hb D-Oak Ridge. All of the variants were found to have the same chemical composition as the 1951 Hb D-Los Angeles variant. This variant results from a point mutation (GAA→CAA) in the beta-globulin gene at the first base of the 121-codon. Consequently, glutamine is produced instead of glutamic acid [11]. Interestingly, Hb D-Punjab is found to have a relatively high incidence in the district of Xinjiang Uygur Autonomous Region of China, and can also be found in certain regions of India, Pakistan, and Turkey [10].

Hb D-Punjab is most commonly inherited in a heterozygous fashion with normal HbA and remains clinically silent. Homozygous inheritance of Hb D-Punjab is rare and usually asymptomatic, however, cases of mild to moderate hemolytic anemia have been reported [12]. Hb D-Punjab can also be inherited with other variants such as β-thalassemia or HbS. When inherited in the presence of β-thalassemia, mild hypochromic and microcytic anemia can occur but is usually clinically silent [13]. In contrast, inheritance of Hb D-Punjab and HbS together can cause vaso-occlusive events including acute chest and stroke, which is similar to homozygous HbS inheritance that manifests clinically as sickle cell disease [9, 14].

Hb Korle-Bu is a rare beta-chain variant that prevails in the population of West Africa. This variant was found to result from a point mutation (GAT→AAT) in the beta-globulin gene at the first base of the 73-codon. Consequently, aspartic acid is produced instead of asparagine [15]. The presence of this variant in the United States is most likely linked to the transatlantic slave trade [16]. When compared to HbS and Hb D-Punjab, very little published research is available on the clinical manifestations of Hb Korle-Bu. In addition, Hb Korle-Bu and HbS migrate at similar lengths on electrophoresis and oftentimes Hb Korle-Bu is misidentified as the more common HbS variant. Hb Korle-Bu and HbS can be better distinguished when electrophoresis is performed with a citrate agar compared to cellulose acetate [17]. Some studies have reported on Hb Korle-Bu in association with HbS and described mild or moderate clinical symptoms but Hb Korle-Bu is typically silent when inherited alone [18, 19].

What is striking about this particular case is that the patient had a combination of Hb D-Punjab, Hb Korle-Bu, and HbS. Per a literature review, it is the only case where a patient had all three variants.

## Conclusions

Hemoglobinopathies have been reported to include a wide range of clinical manifestations like a vaso-occlusive crisis that presented as abdominal pain in this patient. Although our patient was lost to follow up with hematology/oncology to further investigate and identify the genetic mutations involved, it would be important to keep these variants in mind when evaluating patients with suspected hemoglobinopathy. Further investigation and studies, especially in regard to Hb Korle-Bu, would be highly beneficial and are warranted to better characterize the interaction between Hb variants.
